# Comparative Thermo-Mechanical Properties of Sustainable Epoxy Polymer Networks Derived from Linseed Oil

**DOI:** 10.3390/polym14194212

**Published:** 2022-10-08

**Authors:** Madalina Ioana Necolau, Celina Maria Damian, Elena Olaret, Horia Iovu, Brindusa Balanuca

**Affiliations:** 1 Advanced Polymer Materials Group, University Politehnica of Bucharest, 1-7 Gh. Polizu Street, 011061 Bucharest, Romania; 2 Academy of Romanian Scientists, 050044 Bucharest, Romania; 3 Department of Organic Chemistry “C. Nenitescu”, University Politehnica of Bucharest, 1-7 Gh. Polizu Street, 011061 Bucharest, Romania

**Keywords:** bio-based polymers, epoxidized linseed oil, curing reaction, thermal properties, mechanical properties

## Abstract

Considering its great industrial potential, epoxidized linseed oil (ELO) was crosslinked with different agents, both natural and synthetic: citric acid (CA, in the presence of water—W, or tetrahydrofuran—THF, as activator molecules) and Jeffamine D230, respectively, resulting bio-based polymeric matrices, studied further, comparatively, in terms of their properties, through different methods. Thermal curing parameters were established by means of Differential Scanning Calorimetry (DSC). Fourier transform Infrared Spectroscopy (FTIR) and DSC were used to identify the reactivity of each ELO-based formulation, discussing the influence of the employed curing systems under the conversion of the epoxy rings. Then, the obtained bio-based materials were characterized by different methods, establishing the structure–properties relation. Thermogravimetric analysis revealed higher thermal stability for the ELO_CA material when THF was used as an activator. Moreover, a higher glass transition temperature (Tg) with ~12 °C was registered for this material when compared with the one that resulted through the crosslinking of ELO with D230 conventional amine. Other important features, such as crosslink density, storage modulus, mechanical features, and water affinity, were discussed. Under the loop of a comprehensive approach, a set of remarkable properties were obtained for ELO_CA_THF material when compared with the one resulting from the crosslinking of ELO with the synthetic Jeffamine.

## 1. Introduction

Vegetable oils (VO) represent a strong alternative for the synthesis of polymers, at the expense of petroleum-based compounds, being abundant and inexpensive raw materials that can be easily accessed all over the world [1,2]. Renewable and biodegradable characteristics, with low carbon footprint are of great importance [3]. VOs have reached an important industrial demand, being used as raw materials to produce coatings, paints, lubricants, soaps, inks, or polyvinyl chloride plasticizers [4,5,6,7,8]. The common VOs contain fatty acids that vary from 14 to 22 carbons in length, with 0 to 3 double bonds per fatty acid chain. 

Linseed oil (LO), extracted from the seeds of the flax plant, is a drying oil consisting of three types of unsaturated fatty acids: linoleic acid, linolenic acid, and oleic acid. LO has become an important alternative for industrial polymeric materials due to its specific chemical composition, with about 90% unsaturated fatty acid content, based on a branched glycerol core and long aliphatic fatty acid chains which ensure the macromolecule with both strength and flexibility [9]. The higher unsaturation degree (about 6.3 double bonds per triglyceride molecule) offers numerous possibilities for chemical modification to synthesize more complex or more reactive macromolecules. Perhaps the most important and studied reaction at the VOs double bonds is the epoxidation because the oxirane ring gives countless possibilities to obtain high performance materials or it can be subjected to different reaction strategies to further modify the oil structure, obtaining more reactive functionalities grafted on the triglyceride backbone [10]. The epoxidation of LO is preferentially performed by in situ method, using peracetic or performic acids as oxidizing agents, the epoxy content being tailored through the control of the reaction parameters (reactants ratio, time) [10,11]. Aside from all the associated advantages, ELO (like other epoxidized VOs), can lead to polymeric structures through different reaction strategies, but their thermal and mechanical features are not so competitive for industrial applications. In this regard, various research studies were devoted to this subject, especially for the new and more complex crosslinking procedures [11,12].

The current study was conducted starting from the LO converted into epoxidized derivative (ELO) with a high functionalization degree (more than 95%), exploring the possibilities given by the epoxy rings within ELO to obtain bio-based crosslinked polymeric structures with superior properties, with the aid of a sustainable curing agent or a conventional one. Therefore, a better perspective was obtained by using both natural and synthetic curing systems: CA (activated by water—CA_W or tetrahydrofuran—CA_THF) and primary amine species from a commercial polyetheramine (Jeffamine D230), the research being driven as a comparative study of the developed systems. The novelty of the research stays in the use of these systems to crosslink ELO derivatives under the umbrella of the same study and, moreover, the comparative and comprehensive survey of the curing reactions along with the thermal and thermo-mechanical features of the resulted bio-based materials. Thus, the obtained oil-based formulations were investigated to establish their particular reactivity under heating and specific assessments (e.g., DSC), indicating different reaction temperatures for the different designed systems. Further, the resulting ELO-based materials were passed through an ample characterization program by different techniques to establish the fundamental properties of some materials with various industrial applications and the so important structure–properties relation for the envisaged coating application with a high-tech analysis as nanoindentation.

## 2. Materials and Methods

### 2.1. Materials 

LO and all the reagents used for the chemical modification of the triglyceride structure and product purification were purchased from Sigma-Aldrich (Merck KGaA, Darmstadt, Germany). The components of the curing process: citric acid monohydrate (99.5+%) acquired from Alfa Aesar (Thermo Fisher Scientific, Waltham, Massachusetts, United States), anhydrous THF (≥99.9%, inhibitor-free) from Sigma-Aldrich (Merck KGaA, Darmstadt, Germany) and Jeffamine D230, kindly supplied by Huntsman (Texas, United States), were used as received, without any purification. 

### 2.2. Chemical Functionalization of the LO

The epoxidation reaction was performed based on reported procedures for different VOs, using peracetic acid generated in situ [13,14,15]. The resulting product (ELO) was characterized and further used for the bio-based polymer network synthesis. For the ELO monomer, based on the NMR data and previous reports, molecular weight was calculated to be ~ 960 g/ mol and the average functionality was ~ 6 epoxy groups/triglyceride (epoxidation degree of ~96%) [16,17].

### 2.3. Synthesis of the ELO-Based Polymeric Matrices

Both natural and synthetic reagents were tested for the ELO crosslinking reactions: CA and polyetheramine (D230). Due to the reduced solubility of CA in the oily phase, a pre-treatment was required for the CA-based curing systems using two activator molecules, as previously mentioned (W and THF), to identify the suitable crosslinking system for the epoxy derivative. Thus, CA in combination with distilled water represents the *first curing system* studied within the research (H_2_O/-COOH molar ratio = 1/1 [18]), while CA combined with THF is *the second one* (THF/-COOH molar ratio = 4/1 [19]). CA-W and CA-THF mixtures were kept under magnetic stirring at ~90 °C until the complete solubilization of the curing agent, CA. ELO monomer was added to each CA-based curing system with respect to the epoxide/-COOH equivalent ratio of 1/1, observing further the gelation stage of the formulation. *The third curing system* for ELO monomer consists in conventional amine curing agent (Jeffamine D230), 1/1 epoxide/-NH_2_ equivalent ratio being used. The three ELO-based formulations were subjected to different thermal treatments, as shown in Table 1, as resulted from DSC curves onset registered for the initial ELO-based formulations: for the CA-based systems: 80 °C for 3h, 120 °C for 1h and then, a post-curing for 1h at 150 °C. The ELO_D polymeric network resulted through a thermal treatment at 130 °C for 2h, followed by a post-curing stage at 160 °C for 3h.

### 2.4. Characterization Techniques

*Nuclear Magnetic Resonance (NMR) spectra* were registered using a Bruker Advance III HD 600 MHz spectrometer (Bruker, Rheinstetten, Germany), corresponding to the resonance frequency of 600.12 MHz for the ^1^H nucleus, using CDCl_3_ as solvent.

*Fourier Transform Infrared (FTIR) spectra* were registered on a Bruker Vertex 70 equipment in 400–4000 cm^−1^ range with 4 cm^-1^ resolution and 32 scans. The samples were analyzed on ATR module. The conversion of the epoxy groups was calculated for each system from the collected FTIR spectra, using the measured areas (Opus software) of the band in 820–950 cm^−1^ corresponding to the oxiranes and the C=O absorption band, taken as internal reference.

*Differential Scanning Calorimetry (DSC) curves* were recorded on a Netzsch DSC 204 F1 Phoenix equipment. The samples were heated from room temperature (RT) to 300 °C using a heating rate of 10 °C/min under nitrogen (20 mL/min flow rate). From the DSC data, the activation energy (Ea, KJ/mol) was calculated by a simplified method based on Arrhenius equation [20]. Two different temperatures were used for the curing stages, also considering the final conversions of epoxy groups. Applying the logarithm to Arrhenius equation and replacing the reaction rate resulted in Equation (1), used further to calculate the Ea values.

(1) $$\ln\frac{d\alpha }{dt}=\ln k-\frac{E_{a}}{RT},$$

*Thermogravimetric Analysis (TGA)* was performed using a Q500 TA Instruments equipment under nitrogen atmosphere using a heating rate of 10 °C/min from RT to 800 °C. 

*Dynamic-Mechanical Analysis (DMA) tests* were performed on a TRITEC 2000 B equipment. Samples were analyzed in single cantilever bending mode at 1 Hz frequency. Data were collected from −40 °C to 100 °C, using a heating rate of 5 °C/min. Based on the DMA information and reported procedure, crosslink density (mol/m^3^) was calculated for the studied ELO-based materials [21] using the equation:

(2) $$\nu_{e}=\;\frac{E^{\prime }}{3RT},$$

where: E′ = storage modulus at T = Tg + 30 °C, R = gas constant, T = temperature (in K) corresponding to the storage modulus value.

*Contact angle (CA) measurements* were obtained using the Drop Shape Analyzer-DSA100 from Krüss Scientific GmbH (Hamburg, Germany) through static sessile drop method at room temperature using deionized purified water and ethylene glycol (EG) as reference polar and non-polar liquids. The droplet with a volume of 2 μL was maintained on the sample for 5 s. The water contact angle was determined using the Young–Laplace equation in the Advance software and represents the average of three measurements for each sample. Calculation of surface free energies was performed using the same software, which takes into consideration the Young-Dupré and Fowkes equations. Moreover, *water absorption degree (WA)* was measured for all the ELO-based studied samples in accordance with the standard water absorption ASTM D570 method. Shortly, dried and pre-weighted specimens (mi) were entirely immersed in 15 mL tubes containing distilled water and maintained for 7 days (room temperature). Then, the samples were extracted, the water excess was removed by blotting the surfaces and the weights of the swollen samples were then recorded (mf). Measurements were performed in triplicate, the reported values representing the average of the 3 results, calculated according to the Equation 3:

(3) $$WA\;\left(\%\right)=\;\frac{mf-mi\;}{mi}\times \;100,$$

*Mechanical tests* were performed on the Universal Testing Machine (Instron, Model 3382, USA) with 100 kN load-cell at room temperature, using a tensile rate of 10 mm/min. For each studied ELO-based polymeric network, three specimens were tested (1.2 mm thick, 50 mm long and 9.6 mm width). The results obtained from tensile test are shown as stress–strain curves, and data calculation was applied in Bluehill software, giving the Young modulus and elongation at break mean values

*Nanoindentation.* Mechanical properties at microscale were determined through dynamic instrumented indentation using a G200 Nano Indenter system (KLA Instruments) configured with CSM option and a DCM II actuator. All tests were performed at room temperature using a diamond flat-ended cylindrical punch indenter with a punch diameter of 100 µm. All measurements were performed using the implemented “G-Series DCM CSM Flat Punch Complex Modulus” method within NanoSuite software. The employed method returns storage (G′) and loss (G″) modulus over 10 oscillation frequencies ranged between 1 and 110 Hz at 7 µm indentation depth and 50 nm oscillation amplitude. Results are calculated for 0.4 Poisson’s ratio value and are expressed as mean ± standard deviation from 9 different tests (n = 9). 

## 3. Results and Discussion

### 3.1. ELO Synthesis and Structural Characterization

Prior to using it, the ELO product was structurally characterized by means of ^1^H-NMR and FTIR spectroscopy, the obtained spectra being compared to those registered for the crude LO to confirm the conversion of the double bonds of the fatty acids (LO) into epoxy groups (ELO). In the NMR spectrum of ELO derivative, specific signals assigned to the protons of the epoxy rings can be observed at 3.10, 2.95, 1.70, and 1.47 ppm, compared with the spectrum registered for the unmodified oil where can be observed the specific signal at 5.35 ppm belonging to the -HC=CH- protons from the triglyceride double bonds. Moreover, those signals at 2.04 and 2.80 ppm due to the protons near the double bonds (–C**H**_2_–CH=CH_2_ and –CH=CH–C**H**_2_–CH=CH, respectively) are no longer present in the ELO spectrum (Online Resource—Figure S1). 

FTIR analysis confirms this information for ELO product through the specific signals at 820–950 cm^−1^, corresponding to different C-O vibrations of the oxiranes, with a maximum at 825 cm^−1^ (ν_C-O-C_, medium intensity). Moreover, the disappearance of the absorption bands at 3013 cm^−1^ and 1653 cm^−1^ associated with the =C-H and -C=C- stretching vibration, respectively (remarcable in the LO spectrum), indicates the conversion of the LO double bonds from the structure of the fatty acids into epoxy groups (Online Resource—Figure S2).

### 3.2. DSC Studies for the ELO-Based Initial Formulation 

To monitor the curing reactions, DSC analysis was performed for the initial ELO-based formulated systems. A very important aspect to mention is the absence in the DSC thermograms of any crystallization or melting transition of the CA curing agent. This behavior confirms the integration of the CA molecules within the crosslinking system [19].

**Figure 1:**
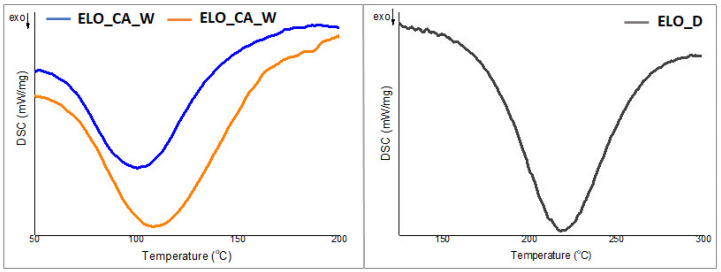
DSC thermograms for the initial bio-based epoxy formulations.

As can be noticed from Figure 1, regardless of the crosslinking system, a single maximum is observed for each ELO-based formulation. The DSC curves can provide insight for the curing protocol, which is required for the chosen systems. In the presence of CA curing agent, the bio-based epoxy networks were formed at a lower temperature as compared with the system crosslinked with the conventional amine, even if the necessary energy for the chemical reactions in the presence of CA is higher, both for water and for the THF activators. As can be observed from Figure 1 and Table 2, the maximum temperature (Tp) for the curing reactions is considerably lower for those systems containing CA. This can be a strong argument for the potential of this curing agent, being important for the synthesis of the sustainable materials starting from the ELO resin. 

The applied curing protocol, at a temperature above 120 °C may lead to β–hydroxyester bonds from the epoxy–acid reaction, which can give unique properties for the ELO type networks in terms of self-healing, relaxation, and certainly superior crosslink density according to a high number of reactive centers [22]. Thus, the broad curve, especially for ELO_CA_THF, may be associated with the formation of these covalent bonds in different reaction stages of the curing process at an elevated temperature due to the residual -COOH groups forming the poly-carboxylic acid and -OH functionalities resulted from the ring opening. The proposed mechanism could be correlated to the calculated high activation energy values (Table 2).

### 3.3. FTIR Spectrometry Studies

**Figure 2:**
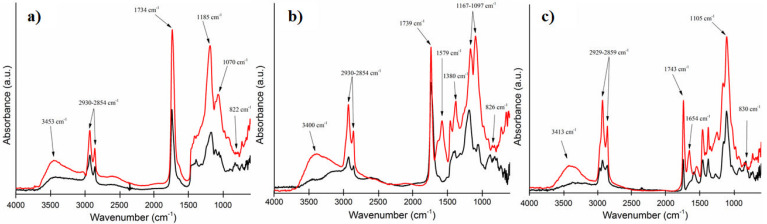
FTIR spectra for the ELO-based systems (initial formulation—black line, final materials—red line): (**a**) ELO_CA_W, (**b**) ELO_CA_THF, (**c**) ELO_D.

To have a large perspective towards the efficiency of each curing system, FTIR spectra for both initial (un-cured) ELO-based systems and the final (cured) matrices were registered (Figure 2).

For the ELO-based formulations, the same conclusion can be highlighted based on the strong diminish (to extinction) of those absorption bands in the spectral range 820–950 cm^-1^, coming from the oxirane groups, which reacted during the curing process, indicating the proper formation of the ELO-derived networks for all the tested crosslinking systems. Moreover, the spectra recorded for the final matrices present increased intensity for the absorption bands between 2850–2950 cm^-1^, associated with ν_C-H_ (CH_2_ groups, C_sp3_), due to the ring-opening reaction. 

For the initial systems containing CA, the wide ν_O-H_ from the carboxylic moieties in the range 3000–3700 cm^−1^ can be easily recognized. Contrarily, for the cured materials, the presence of the -OH characteristic absorption maxima around 3400 cm^−1^ confirms the COOH-epoxide reaction along with the network formation by the epoxy ring opening. For the ELO_CA_THF cured material, a new signal can be observed at 1579 cm^−1^. This peak can be associated with residual carboxylate anion coming from CA crosslinker [23]. For both ELO_CA_W and ELO_CA_THF some evidence of esterification reactions can be observed, highlighted by those signals in the spectral range 1070–1185 cm^−1^ (ν_C-O_). For ELO_CA_W, the signal at 1170 cm^−1^ (ν_C-O_ asymmetric stretch) splits in two maxima (1070 cm^−1^ and 1185 cm^−1^) and, in the same way for the ELO_CA_THF system, the ester band from 1194 cm^−1^ splits in two maxima (1097 cm^−1^ and 1167 cm^−1^) as a consequence of the formation of new types of covalent C-O-C bonds. The peaks at 1070 cm^−1^ and 1097 cm^−1^ are representative of the ether asymmetric stretch band with respect to the epoxy–acid curing mechanism [23]. All these findings confirm the reaction of the -COOH moieties from CA with the epoxy rings from ELO. 

### 3.4. The Curing Kinetics for the Formulated ELO-Based Systems

**Figure 3:**
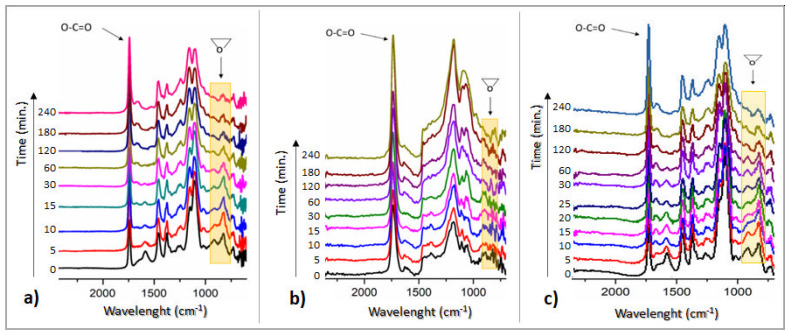
FTIR spectra from the curing kinetics of the ELO-based systems at different reaction times: (**a**) ELO_CA_W at 80 °C, (**b**) ELO_CA_THF at 80 °C, (**c**) ELO_D at 130 °C.

The structural changes that occur during the thermal curing reactions performed for the bio-based epoxy systems were monitored by FTIR spectrometry. The curing kinetics were monitored at two different temperatures: 80 and 100 °C for CA-crosslinked systems and 130 and 160 °C for ELO_D system, at different reaction times, based on the evolution of specific absorbance bands corresponding to the molecular vibrations of the epoxy rings (820–950 cm^−1^), consumed during the thermal curing. The extent of the reaction was calculated by taking as reference the absorption band located around 1740 cm^−1^, corresponding to the ester carbonyl in the triglyceride moiety. Figure 3 presents the FTIR spectra recorded at different reaction times for selected reaction temperatures. 

From the obtained spectral data (Figure 3), there can be observed that ELO-based epoxy networks reached an almost full conversion of the oxirane groups in the selected reaction conditions. The reaction of the ELO epoxides under temperature seems to proceed faster for the two systems containing CA as a crosslinker, considering the thermal curing protocol developed based on the initial DSC curves.

**Figure 4:**
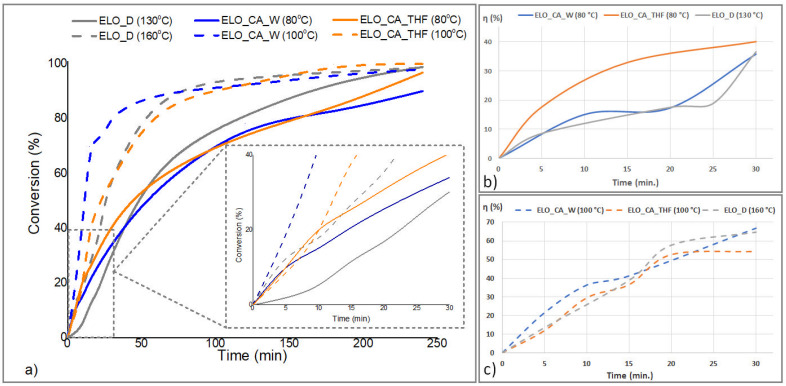
Curing kinetics calculated for ELO-based systems by DSC at different temperatures (**a**) and from the FTIR spectra in the first 30 min. of reaction, at lower temperatures (**b**) and higher temperatures (**c**).

Examining the FTIR conversion vs. time graph at lower temperatures (Figure 4b), in the initial stage, ELO_CA_THF seems to be the most reactive systems, probably due to the great efficiency of the crosslink constituents with low-molecular activator, compared to the ELO molecule, and great compatibility of THF with the oily monomer. Moreover, t is possible for the temperature-sensitive THF molecules to reach an excited state that successfully activates the citric acid molecule, resulting in the rapid start of the reaction with the epoxy rings on the ELO structure. Conversely, when the curing kinetic was monitored at a higher temperature, this system no longer seemed to be the most reactive, but the water-activated one took its place. Again, this could be attributed to the low molecule volume along with the increased temperature, which may produce a proper CA activation. These findings are in good agreement with the epoxy curing kinetic theory, the beginning of the reaction being temperature dependent [24].

As the reaction progresses over time, the ELO_CA_W system registered a great curing rate (83.7%, calculated from the FTIR spectrum registered at 180 min. of reaction), the diffusion of the activating small molecule being unrestricted by the concurrent reaction of crosslinking. The end of the crosslinking process at the highest temperatures brings reaction yields over 90% for all the studied systems. 

The reaction kinetics were also monitored by DSC using the same temperature conditions: 80 and 100 °C for ELO_CA systems and 130 and 160 °C for ELO crosslinked with D230. The first crosslinking stage was temperature dependent, governed by the molecular moving and the activity of the functional groups, and facilitating the interactions with the epoxy groups from ELO. In this context, water has the highest dynamic in the ELO system, as can be seen from Figure 4a-inset, these results being well correlated to the previously discussed ones. The second step is correlated to the diffusion process of the active species, ELO_CA systems being the most reactive. For the ELO_D system, a clear induction period was observed, given by the lower mobility of large polyether molecules. Relating to the FTIR kinetic results, the difficult diffusion of D230 molecules among the ELO fatty acid chains to the epoxy active centers was also observed, especially in the initial stage and at the lowest temperature (130 °C). 

### 3.5. Thermal Stability

**Figure 5:**
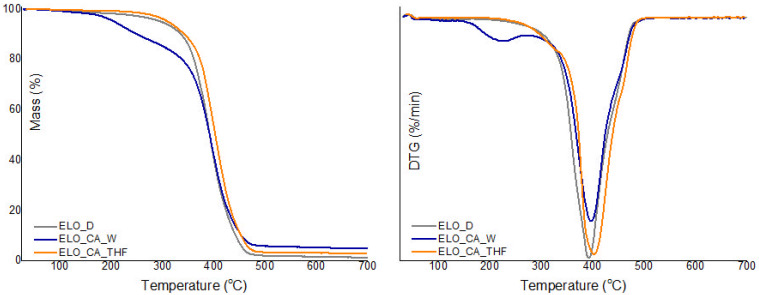
(**left**) TGA and (**right**) DTG curves of the studied bio-based epoxy matrices.

Thermal stability profiles of the bio-based epoxy matrices were established by TGA; the obtained results are depicted in Figure 5. Regarding the thermal stability of the ELO-based networks, quite different degradation profiles were registered according to the variation of the chemical agents used as a crosslinker for the ELO resin. 

When THF was used as an activator for the crosslinking agent, the resulting material stood out in terms of thermal stability, as can also be noticed from Table 3, which is a consequence of intermolecular H bonding, along with the efficient formation of β–hydroxyester linkages through the reaction of the epoxy rings with the CA functional groups, leading to a compact bio-based epoxy network.

The low thermal stability registered for the ELO_CA_W system may be a consequence of the high amount of linear aliphatic moieties along with thermally cleavable ester bonds; this type of linkages is more susceptible to tensioned conformation in comparison with the polyether chains, characteristic for epoxy–amine crosslinking. Moreover, a possible lower efficiency of the crosslinking reaction associated with this system, leading to low molecular weight oligomeric fraction easily degradable under temperature along with hydroxyl groups which are susceptible to moisture representing the principal reasons for the registered lower thermal stability [19].

Usually, conventional systems have stood out over time as candidates which lead to crosslinked epoxy matrices with high thermal stability [25], but when synthetic Jeffamine was employed to crosslink ELO, resulting ELO_D material is unremarkable from this point of view, registering a Td5% value below 300 °C. 

The first degradation stage (150–300 °C) may be the result of the thermal decomposition of the partially crosslinked epoxidized oil and also the residual solvents incorporated in the curing systems. The major degradation stage in the temperature range 350–460 °C is attributed to the ester groups scission from the ELO [26], while, at elevated temperatures (above 430 °C), those ether and ester bonds established through the crosslink reactions between poly-carboxylic acid and epoxides from the oil monomer are broken. When the temperature increases above 500 °C, almost full degradation of the ELO-based materials can be observed, with a low quantity of residual contents (Table 3). 

When DTG curves are analyzed, a similar degradation profile can be observed for all the studied materials, with one decomposition stage in the temperature interval 394–403 °C. ELO_CA_W registered an auxiliary decomposition maxima around 200 °C that may be associated to a fraction of low molecular weight or uncured fragments caught within the network; these fragments may occur as a consequence of the heterogeneous triglyceride structure or steric hindrance affecting the epoxy ring-opening reactions in the presence of CA_W system. It is premature to state a general conclusion, but also, from the thermal stability point of view, ELO-CA_THF seems to be a promising material.

### 3.6. Dynamic-Mechanical Analysis 

Important information regarding network flexibility along with crosslink density is pointed out by DMA. Every studied system shows a particular behavior for both Tg and crosslink density, as a result of the used bio-based monomer, curing agent or the activator. 

Regarding the glass transition (Table 3, Online Resource—Figure S3), the higher Tg of 41 °C was registered for ELO crosslinked with CA-THF, situated within the usual limits for the crosslinked epoxy resins derived from vegetable oils [14]. As indicated by the DSC study, this system tends to form supplementary covalent bonds between the system components (residual -COOH groups from CA and -OH groups from the ELO ring opening), also reflected in the calculated crosslink density value, THF proving to be an effective activating agent for poly-carboxylic acid used in the synthesis of these bio-based materials. Instead, the lower crosslink density was calculated for the ELO cured with the synthetic Jeffamine, being well known that the crosslink density is a factor strongly influenced by the functionality of the molecule used as a crosslinker [27]. Thus, the CA leads to higher crosslink density by the three -COOH functionalities from its structure and especially when THF is used to activate the acid molecule; at an elevated temperature, a large number of new ester groups are formed, as FTIR spectrometry already confirms. 

**Figure 6:**
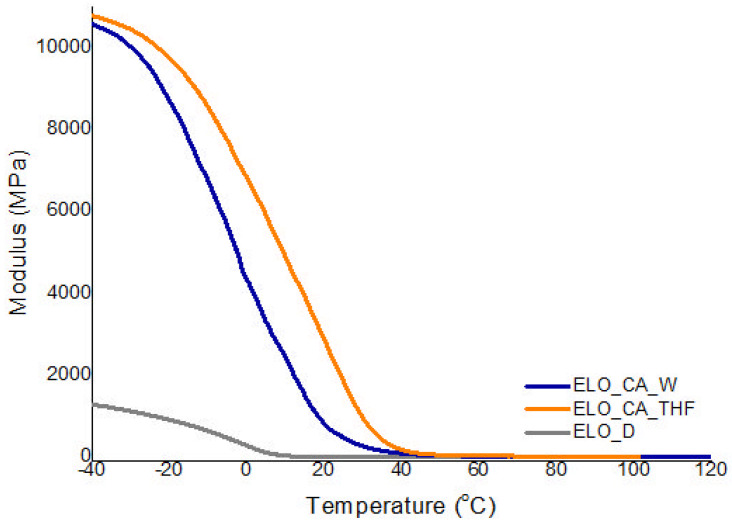
Variation of the storage modulus with the temperature for the ELO-based matrices.

The storage modulus is an important component of viscoelastic behavior which characterize the bio-epoxy networks from a stored energy point of view. Thus, by using DMA, it was observed that ELO_CA_THF systems displayed the highest value for the modulus over the temperature range (Figure 6), as compared with the other studied systems, which is correlated with a high energy demand over sample deformation. However, storage modulus values for ELO_CA_W are slightly lower, and they can be correlated with the increase in intermolecular H bonding. The ELO_D sample is the most flexible one (low storage modulus) due to the polyether chains, which are large molecules, thus conducting to a higher freedom degree within the epoxy network. This feature is well correlated to the registered Tg values of the ELO-based materials.

### 3.7. Nanoindentation

Storage modulus (G’), known as the elastic component of modulus and loss modulus (G”) or viscous constituent of modulus, were evaluated through dynamic instrumented indentation, as described previously [28,29,30] since epoxidized vegetable oil-based polymers are highly compliant materials. The obtained results (plotted in the Online Resource—Figure S4) show different behaviors for the tested samples. While ELO_CA_THF exhibit an elastic behavior dependent on the applied frequency with G’ exceeding G″, the ELO_D sample shows both elastic and viscous behaviors with G’ higher than G” for the most part of the frequency range. However, the ELO_CA_W sample exhibited both types of behaviors independent of the frequency studied range, with G″ slightly higher than G’ almost over the entire frequency interval.

**Figure 7:**
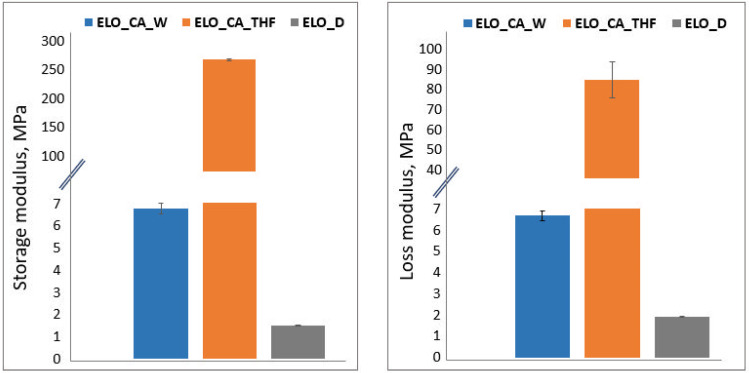
Storage and Loss Modulus data obtained through nanoindentation technique at 1 Hz.

The higher crosslink degree within the bio-epoxy matrix correlated with a high storage modulus leads to a reduced elastic behavior. The low elasticity is a consequence of the restricted chains motion and a reduced free volume within the well-crosslinked network (short chain segments between crosslinking points), the higher G’ values observed for the ELO_CA_THF system being thus explained. The nanoindentation results (Figure 7) are in accordance with the DMA results, where it was observed that for the CA-cured systems the elastic behavior becomes dominant (in the glassy state), whereas the influence of the viscous behavior is diminished. Low values obtained for both G’ and G″ components corresponding to ELO_D system may be associated with the viscoelasticity of the crosslinked ELO, this behavior being a known problem in the indentation assays of the soft coatings, where liquid-like nature is dominant [31,32].

### 3.8. Mechanical Test

**Figure 8:**
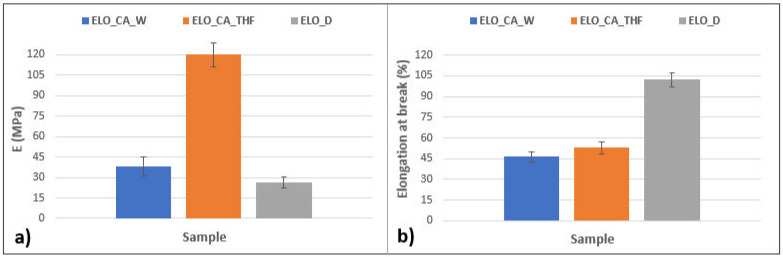
Mechanical test results for the ELO-based polymeric networks: (**a**) Young modulus (MPa), (**b**) elongation at break (%).

The calculated Young modulus (graphical representation in Figure 8) clearly indicates the great elongation resistance of the ELO_CA_THF polymeric material. This finding is well correlated to the calculated crosslink density, the higher number of the reaction points within the network, and the higher resistance of the material under the tensile tests. The ELO_D system seems to exhibit the lowest resistance on the tensile experiments, correlating to the calculated crosslink density value. Once again, the CA-THF system demonstrates its great potential as a crosslinker for the epoxidized vegetable oil, leading to high-performant materials in terms of mechanical properties when compared to the other curing system, CA-W, or to the conventional Jeffamine D230. 

**Figure 9:**
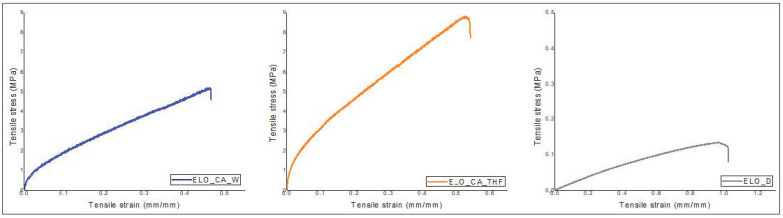
Graphical representation of stress–strain curves for ELO-based polymeric materials.

The mechanical properties of the studied epoxy networks were also investigated, and the stress–strain relationship was depicted in Figure 9. The obtained results are well correlated with those findings given by other analyses. The maximum strength shows that ELO_CA_THF has a compact network translated in the best value of tensile stress (8.79 MPa). This is an improvement of 68% gained by only using a different activation molecule for the CA curing agent. Although, the systems crosslinked with conventional amine have lower strength; the tensile strain and accordingly the elongation at break are higher than ELO-CA type systems due to the molecular flexibility of the Jeffamine chains, as it was stated from DMA and nanoindentation assays. In this context, the values obtained for elongation at break validate the crosslink density ones.

### 3.9. Degradation Studies

Curing at a temperature above 120 °C encourages the formation of β-hydroxyester linkages, which can be easily broken by alkaline solutions through a hydrolytic mechanism [33]. This can be considered an advantage against conventional epoxy resins, which cannot be easily recycled. Thus, to identify the degradability of the bio-based ELO-based system, degradation assays were performed for the oil-based matrices cured with both CA and D230.

After 24 h at room temperature in NaOH solution (50% wt.), both ELO_CA_W and ELO_CA_THF totally degraded (images in Online Resource—Figure S5). Conversely, ELO_D material was not affected by the aggressive alkaline medium.

### 3.10. Wettability Assessment

**Figure 10:**
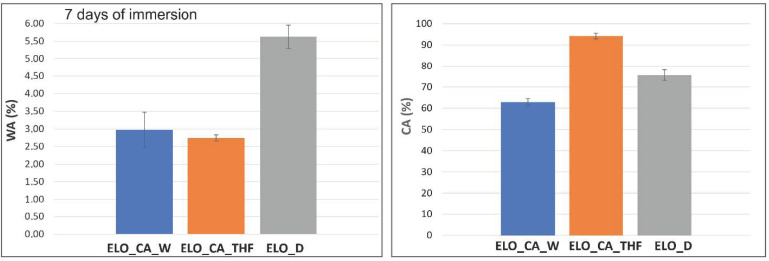
Graphical representation of measured WA (%) and CA (°). For the studied ELO-based crosslinked networks.

The wettability of the ELO-based matrices was investigated by contact angle measurement, using the Young–Laplace fitting method and water absorption degree (WA, calculated according to ASTM D570 method), the results being presented in Table 4 and Figure 10. 

The increased surface hydrophobicity associated with the measured CA values of ELO_CA_THF can be attributed to a higher number of H bonds which can occur within the crosslinked ELO polymer and moreover to the possibility stated above regarding supplementary crosslinking points formed between residual -COOH functionalities form the crosslinker and -OH groups resulted from the ring opening, respectively, conducting to a more packed network associated to a higher crosslink density calculated for this system. In this case, the THF activator proved again to be the more efficient for the crosslinking agent when compared with the curing system formed by citric acid and water. The contact angle value registered for ELO_D matrix situated between the ELO_CA_THF and ELO_CA_W measured values can be explained by the Jeffamine chemical nature, more hydrophobic than the tricarboxylic acid.

The bulk water affinity was also determined, WA degree being gravimetrically calculated and graphically represented in Figure 10. It can be observed that ELO_CA_THF material is the more hydrophobic one, in accordance with the CA measurement, registering a WA of less than 3% after seven days of immersion. Moreover, the low surface energy value is correlated with the hydrophobic character of this bio-based epoxy network, this behavior being associated with a compact and well-crosslinked material, as also indicated by DSC and DMA studies. On the other hand, the WA value calculated for ELO cured with Jeffanime D230 is higher than those for citric acid-based systems, in accordance with the crosslinker molecular volume, inducing a higher bulk water affinity (~5.5%). This behavior indicates a specific and different arrangement of the oil phase on the surface material and in the mass materials, respectively. When THF is used in ELO system, the H bonding leads to a more packed structure associated with high CA value and low WA degree (hydrophobic behavior), while D230 intermediates the surface hydrophilicity but also water penetration within the bulk material, probably due to the unshared electrons from the C-O bonds able to link with the immersion medium. 

The obtained results are strong arguments for the tricarboxylic acid ability to conduce to well-crosslinked bio-based materials, especially when THF is used as activator, registering superior hydrophobicity, when compared to other studied systems [34].

## 4. Conclusions

The research was conducted starting from linseed oil used to synthesize the epoxy monomer (ELO), to the fabrication of three types of ELO-based formulations with different crosslinking systems: citric acid (with water or THF as activating molecules) or conventional Jeffamine D230, evaluating the main properties of the materials obtained by thermal curing. 

The efficacy of the used thermal curing protocols was confirmed by FTIR spectrometry and DSC reaction kinetics. Among the studied systems, the one obtained by using THF as an activator stands out by its reactivity due to good compatibility with the oil-based system, leading to great efficiency in the ELO crosslinking process. Moreover, water as the activating molecule for the CA crosslinking agent indicates a very good efficiency regarding ELO curing reaction given by a final cure yield of over 90%. 

The thermal degradation of the system containing CA-THF indicates the highest potential, showing a T_d5%_ value of ~33% higher and a T_d10%_ value of ~27% higher than ELO cured with CA-W. Better thermal stability, compared with the degradation profile registered by the ELO_D polymeric matrix, was also noticed. These results are well correlated with the calculated crosslink density from DMA data. The results are sustained by the hypothesis expressed as a possible supplementary reaction between the residual -COOH groups form the poly-carboxylic acid and the -OH groups resulting from the epoxy ring opening. Such a reaction mechanism is also supported by DSC on the initially formulated systems and the calculated activation energy. 

Mechanical and thermo-mechanical results investigated by means of DMA, nanoindentation, and tensile test also indicated a great potential for the ELO_CA_THF bio-based polymeric matrix due to its higher crosslink degree correlated with a high storage modulus compared to the other studied systems.

All the investigated ELO-based materials indicated low water affinity after seven days of immersion, with ELO_CA_THF recording an absorption degree below 3% after this time. Contact angle values over 90° for this system are in accordance with the above findings. This performance may be attributed to barrier properties against water exerted by the ELO network, efficiently crosslinked by the CA-THF. All these results underline the great potential of these materials in technical and industrial applications, such as protective coatings for different substrates or encapsulation systems for electronics.

## Figures and Tables

**Table 1:** ELO-based formulations and the used curing parameters.

Polymer Matrix					
System Code	Components				Curing Temperatures
	ELO	CA		D230	
		H_2_O	THF		
ELO_CA_W	x	x	-	-	80 °C, 3 h 120 °C, 1 h 150 °C, 1 h
ELO_CA_THF	x	-	x	-	80 °C, 3 h 120 °C, 1 h 150 °C, 1 h
ELO_D	x	-	-	x	130 °C, 2 h 160 °C, 3 h

**Table 2:** DSC and GF results for ELO-based matrices.

Sample	∆H ^a^ (J/g)	Tp ^b^ (°C)	Ea ^c^ (KJ/mol)
ELO_CA_W	125.5	100.4	39.27
ELO_CA_THF	174.1	108.0	69.84
ELO_D	170.7	218.4	20.04

^a^—curing enthalpy calculated from polymerization peak area; ^b^—maximum temperature of polymerization peak calculated from inflection of cooling curve; ^c^—activation energy.

**Table 3:** TGA and DMA data for the ELO-based synthesized materials.

Sample	TGA				DMA	
	T_d5%_^a^ (°C)	T_d10%_^b^ (°C)	Residual Mass (%, at 700 °C)	T_max_ (°C)	Tg ^c^ (°C)	Crosslink Density (mol/ m^3^)
ELO_CA_W	206.9	250.7	4.94	395.8	39	1414
ELO_CA_THF	311.4	343.0	3.07	402.4	41	2477
ELO_D	298.2	336.2	1.36	394.5	29	417

^a^/^b^—temperature at which the degradation is 5/10%, respectively; ^c^—glass transition temperature considered as the maximum of tanδ plots.

**Table 4:** CA results for the ELO-Based samples.

Sample	θ (°, water)	θ (°, EG) ^a^	Surface Free Energy (Nm/m)
ELO_CA_W	62.89 ± 1.65	52.37 ± 2.54	41.50
ELO_CA_THF	94.25 ± 1.36	67.49 ± 1.53	29.50
ELO_D	75.65 ± 2.45	61.73 ± 2.48	28.39

^a^—EG = ethylene glycol.

## Data Availability

Not applicable.

## Supplementary Materials

The following supporting information can be downloaded at: https://www.mdpi.com/article/10.3390/polym14194212/s1, Figure S1: ^1^H-NMR spectra of crude LO and ELO derivative; Figure S2: FTIR spectra of unmodified oil (a) and ELO derivative (b); Figure S3: Tan delta vs. temperature graphs for the ELO-based materials; Figure S4: G” (square) and G’ (circle) progress related to frequency for ELO-based matrices; Figure S5: Behavior of the ELO-based materials in NaOH (50% solution) (initial and after 24 h immersion).
