# Supplementary material for: Comparative Thermo-Mechanical Properties of Sustainable Epoxy Polymer Networks Derived from Linseed Oil
Source: Polymers (Basel). 2022 Oct 8;14(19):4212. doi: 10.3390/polym14194212 (PMC9570653; doi:10.3390/polym14194212)
Supplement: Supplementary file 1 [file polymers-14-04212-s001.zip › polymers-1931762-supplementary.pdf]

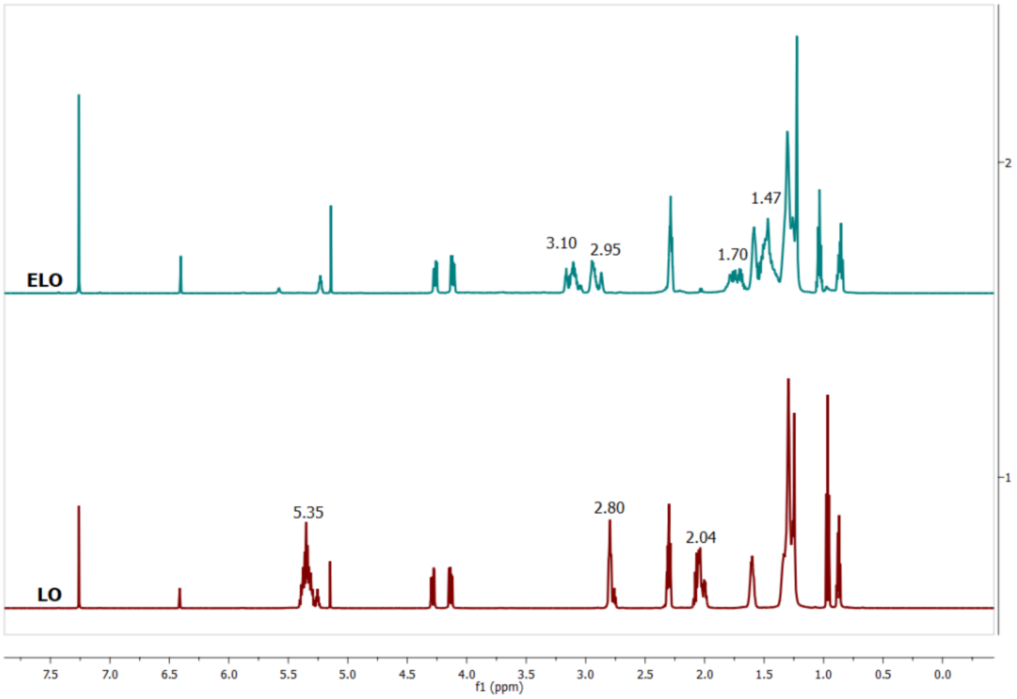

Figure S1. ^{1}H-NMR spectra of crude LO and ELO derivative.

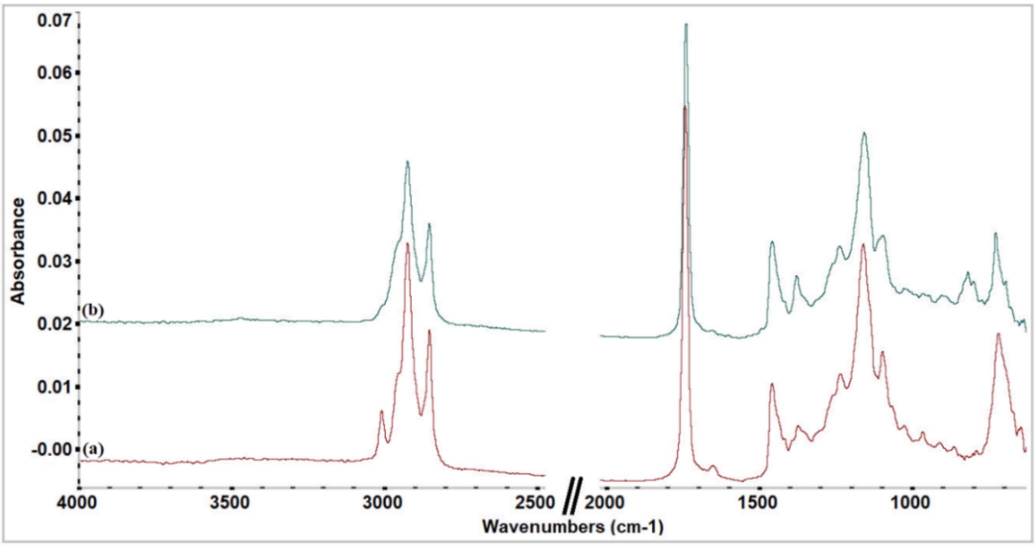

Figure S2. FTIR spectra of unmodified oil (a) and ELO derivative (b).

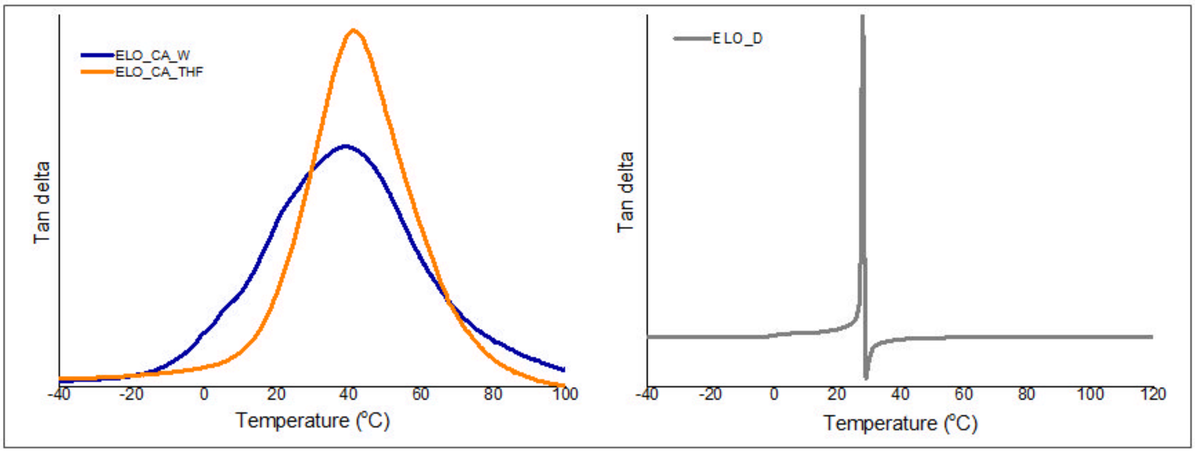

Figure S3. Tan delta vs. temperature graphs for the ELO-based materials.

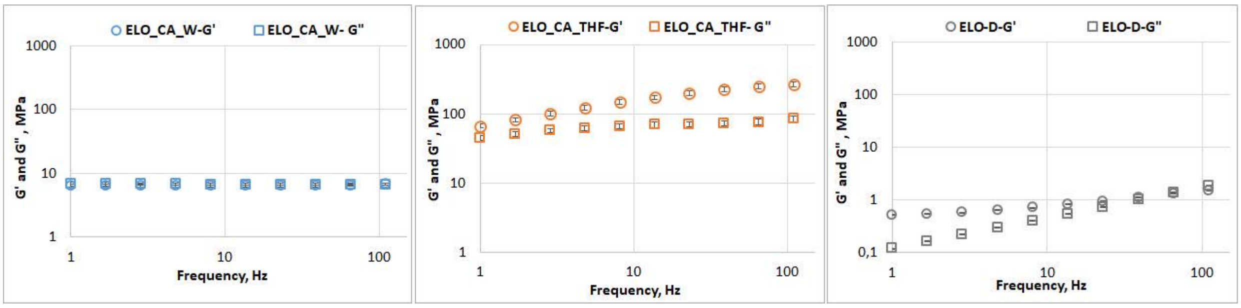

Figure S4. G″ (square) and G′ (circle) progress related to frequency for ELO-based matrices.

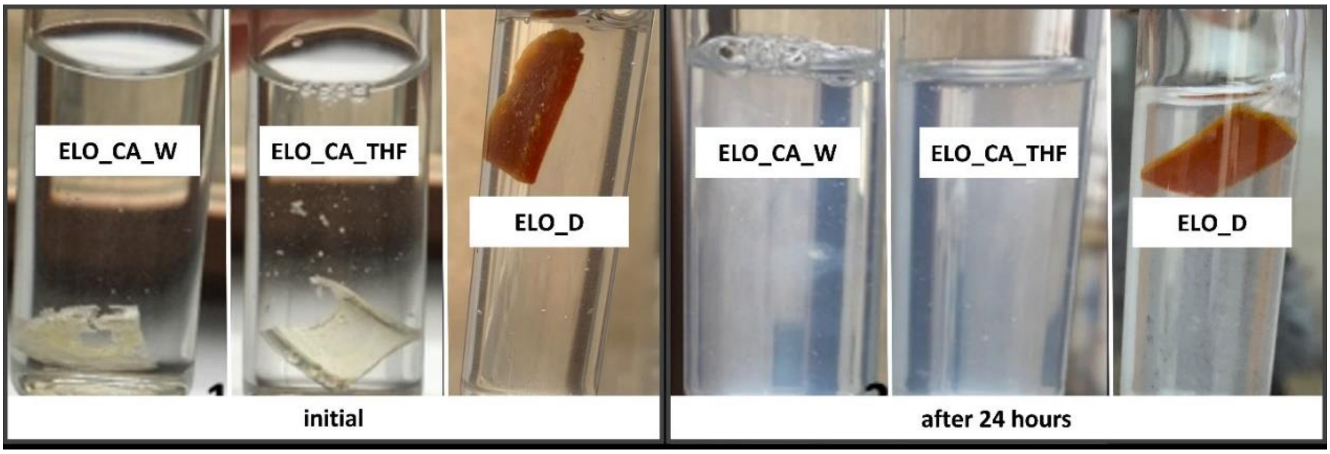

Figure S5. Behavior of the ELO-based materials in NaOH (50% solution) (initial and after 24h immersion).
